# Fluid Selection in Renal Transplant Patients: Considerations for Hyperkalemia Management

**DOI:** 10.4274/TJAR.2025.251963

**Published:** 2025-05-30

**Authors:** Furkan Tontu

**Affiliations:** 1 University of Health Sciences Türkiye Başakşehir Çam and Sakura City Hospital, Clinic of Anaesthesiology and Reanimation, İstanbul, Türkiye

**Keywords:** Acid-base balance, balanced crystalloids, fluid selection, hyperkalemia management, perioperative care, renal transplantation

## Dear Editor,

I would like to extend my gratitude to the authors for their comprehensive and valuable review on this important topic.^1^ Recognizing that appropriate fluid selection may significantly impact clinical outcomes and enhance recovery after surgery (ERAS) in renal transplant recipients, I seek to contribute to their review by providing a more detailed analysis of crystalloid selection.

**Figure 1:**
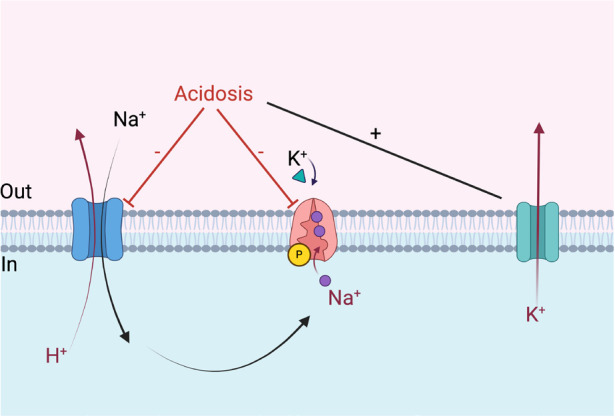
Effects of acidosis on Na⁺-H⁺ exchange, Na⁺-K⁺ ATPase pump, and K⁺ efflux in a muscle cell [Created in BioRender. Tontu F. (2025) https://BioRender.com/z88e118]

Metabolic acidosis occurs in the progression of chronic kidney disease as a result of reduced acid excretion capacity combined with increased daily endogenous and exogenous acid production. However, the hyperkalemia observed in these patients is a consequence, not a cause, of metabolic acidosis.^2^ And since hyperkalemia is a common challenge in renal transplant patients, clinicians have traditionally favored 0.9% saline infusion due to its lack of potassium. Saline-induced hyperchloremia reduces the strong ion difference, which promotes water dissociation and consequently leads to metabolic acidosis.^3^ Acidosis inhibits Na⁺-H⁺ exchange, one of the key pathways maintaining pH and potassium homeostasis in cells, thereby increasing potassium efflux (see Figure 1).^4^ Since 98% of total body potassium resides intracellularly, the disruption of K⁺ homeostasis between intracellular and extracellular compartments (e.g., due to saline-induced acidosis) is considered a more critical factor than external potassium intake (e.g., potassium-containing balanced crystalloids).^2^ Furthermore, high-chloride solutions, such as saline, may induce renal vasoconstriction and lead to a reduction in glomerular filtration rate.^5^ This has led some clinicians to prefer potassium-containing solutions with chloride concentrations more comparable to plasma, such as Plasma-Lyte and Ringer’s lactate (RL). The authors state in their review that “The proper fluid type remains controversial and open for research, but balanced crystalloid solutions seem to be the best choice” and cite two references to support this claim.^1^^, ^^6^^, ^^7^ However, neither of these references discusses the superiority of one crystalloid type over another. One reference merely suggests that the use of crystalloids is appropriate but does not specify which type of crystalloid should be preferred.^6^ The other reference reveals an association between overhydration, microinflammation, and endothelial dysfunction, without addressing fluid type selection.^7^ Yet, numerous studies have demonstrated the advantages of balanced crystalloids over saline in renal transplant recipients. Studies comparing RL and saline in renal transplant patients have shown that potassium concentrations in the RL group are similar to or even lower than those in the saline group.^8, 9, 10^ Studies comparing Plasma-Lyte with saline have reported a higher incidence of hyperkalemia, hyperchloremia, and metabolic acidosis in the saline group.^11, 12, 13^ Additionally, better diuresis and less frequent use of renal replacement therapy early after surgery, as well as better graft function at 3 months, were observed in the Plasmalyte group, which may also contribute to ERAS protocols.^11^ The American Society of Anesthesiologists Transplantation Committee has stated that, in renal transplant patients, perioperative balanced crystalloid solutions are associated with a better metabolic profile and comparable or lower potassium levels compared to saline. Therefore, their use is recommended (GRADE moderate-quality of evidence, strong recommendation).^14^ The 2016 Cochrane meta-analysis reported that balanced solutions are associated with a lower incidence of hyperchloremic metabolic acidosis compared to saline; however, the impact of low-chloride solutions on graft outcomes remains uncertain.^15^ However, the BEST-Fluids trial, a multicenter, double-blind, randomized, controlled study published in 2023, yielded striking findings regarding delayed graft function (DGF) in renal transplant patients.^16^ The study included 807 patients who underwent deceased donor kidney transplantation, randomized into two groups: saline (n = 403) and balanced crystalloid (n = 404). DGF was observed in 120 patients (30%) in the balanced crystalloid group, compared to 160 patients (40%) in the saline group [adjusted relative risk 0.74 (95% confidence interval 0.66-0.84; *P*<0.0001)]. Based on these findings, the use of balanced crystalloids in deceased donor kidney transplant recipients has been emphasized as the standard of care.^16^ In early 2025, a highly recent meta-analysis incorporating data from 10 studies and 1,532 patients was published.^17^ According to this analysis, balanced crystalloids significantly reduce the risk of DGF compared to saline in deceased donor kidney transplantation. However, no significant difference was observed between the two groups regarding the risk of hyperkalemia or DGF in living donor kidney transplantation.^17^ The authors suggest that targeted fluid therapy and optimized perioperative hemodynamic management can prevent DGF.^1^ Additionally, the selection of a crystalloid type may also be crucial in preventing DGF and contributing to ERAS protocols.

In summary, the current literature demonstrates that balanced crystalloids offer several advantages over saline, which may also contribute to ERAS protocols, without posing an additional risk of hyperkalemia.
